# Anti-TNF- *α*treatment-related pathways and biomarkers revealed by transcriptome analysis in Chinese psoriasis patients

**DOI:** 10.1186/s12918-019-0698-7

**Published:** 2019-04-05

**Authors:** Lunfei Liu, Wenting Liu, Yuxin Zheng, Jisu Chen, Jiong Zhou, Huatuo Dai, Suiqing Cai, Jianjun Liu, Min Zheng, Yunqing Ren

**Affiliations:** 1 Department of Dermatology, Second Affiliated Hospital, Zhejiang University School of Medicine, Hangzhou, China; 2 School of Public Health and Management, Hubei University of Medicine, Shiyan, Hubei, China; 3 Human Genetics, Genome Institute of Singapore, Singapore, Singapore; 4 Second Clinical Medical College, Zhejiang Chinese Medical University, Hangzhou, China

**Keywords:** Psoriasis, Anti-tumor necrosis factor alpha (TNF- *α*) treatment, Etanercept, Peripheral blood mononuclear cells (PBMCs)

## Abstract

**Background:**

Anti-tumor necrosis factor alpha (TNF- *α*) therapy has made a significant impact on treating psoriasis. Despite these agents being designed to block TNF- *α* activity, their mechanism of action in the remission of psoriasis is still not fully understood at the molecular level.

**Results:**

To better understand the molecular mechanisms of Anti-TNF- *α* therapy, we analysed the global gene expression profile (using mRNA microarray) in peripheral blood mononuclear cells (PBMCs) that were collected from 6 psoriasis patients before and 12 weeks after the treatment of etanercept. First, we identified 176 differentially expressed genes (DEGs) before and after treatment by using paired t-test. Then, we constructed the gene co-expression modules by weighted correlation network analysis (WGCNA), and 22 co-expression modules were found to be significantly correlated with treatment response. Of these 176 DEGs, 79 DEGs (M_DEGs) were the members of these 22 co-expression modules. Of the 287 GO functional processes and pathways that were enriched for these 79 M_DEGs, we identified 30 pathways whose overall gene expression activities were significantly correlated with treatment response. Of the original 176 DEGs, 19 (GO_DEGs) were found to be the members of these 30 pathways, whose expression profiles showed clear discrimination before and after treatment. As expected, of the biological processes and functionalities implicated by these 30 treatment response-related pathways, the inflammation and immune response was the top pathway in response to etanercept treatment, and some known TNF- *α* related pathways, such as molting cycle process, hair cycle process, skin epidermis development, regulation of hair follicle development, were implicated. Furthermore, additional novel pathways were also suggested, such as heparan sulfate proteoglycan metabolic process, vascular endothelial growth factor production, whose transcriptional regulation may mediate the response to etanercept treatment.

**Conclusion:**

Through global gene expression analysis in PBMC of psoriasis patient and subsequent co-expression module based pathway analyses, we have identified a group of functionally coherent and differentially expressed genes (DEGs) and related pathways, which has not only provided new biological insight about the molecular mechanism of anti-TNF- *α* treatment, but also identified several genes whose expression profiles can be used as potential biomarkers for anti-TNF- *α* treatment response in psoriasis.

**Electronic supplementary material:**

The online version of this article (10.1186/s12918-019-0698-7) contains supplementary material, which is available to authorized users.

## Background

Psoriasis is a common immune-mediated chronic inflammatory skin disease affecting up to 3% of the world’s population [1]. It is characterized by the marked hyperproliferation of keratinocytes, infiltration of immune cells and vascular remodeling in the skin. Approximately a third of patients have a moderate-to-severe form of the disease, and have joint involvement, which has severely negative effect in the patient quality of life.

The immunopathogenesis of psoriasis is thought to be a complex interplay between T cells, dentric cells and keratinocytes, which generates the inflammatory process in the skin [2]. Biological agents (Etanercept, Infliximab and adalimumab) targeting TNF- *α* have showed high efficacy in psoriasis therapy, which consists with the pivotal role of TNF- *α* in the pathogenesis of psoriasis. As a pro-inflammatory cytokine, TNF- *α* possesses multiple activities in the development and maintenance of psoriasis, including recruiting T cells to the skin and increasing the proliferation of keratinocytes. It has been shown that TNF- *α* level significantly increased in all layers of the epidermis, dermal blood vessels in lesional skin, in the blood serum, and the synovial fluid of patients with psoriatic arthritis comparing with healthy controls [3].

Inhibition of TNF- *α* is known to neutralizes the biological function of this cytokine by blocking its interaction with the cell surface TNF-a receptors, but the anti-inflammatory mechanisms of anti-TNF- *α* are still not fully understood. Recently, a few studies have been conducted to elucidate the mechanism of action of these anti-TNF agents in the resolution of psoriasis, focusing on different gene expression in psoriatic lesion and peripheral blood.

Skin samples from patients with anti-TNF-a treatment have been shown to downregulate Th17 [4, 5] and IL12/23 [6] -driven immune response in lesional psoriatic skin. Johnston et al. [7] further provided evidence that early responses of psoriasis to etanercept may be due to decrease tissue responsiveness to IL-17A as a result of suppressed IL-17RC expression in keratinocytes. In addition, the association with clinical remission induced by anti-TNF treatment was also described between inhibition of IL-20 subfamily [8], CCR7/CCL1 [9] axis and p38 mitogen-activated protein kinase (MAPK) activity [10] in skin lesions. In another study, Bosè et al. [11] reported a dual role of anti-TNF- *α* therapy by inhibiting the expression of Th17/Th1 cytokine and early inflammatory genes in the skin whilst enhancing TCR-mediated Th17/Th1 cell activation in peripheral blood. Chow et al. [12] also demonstrated the different effects of adalimumab on gene expression, finding that pathways modulating the differentiation and proliferation of epidermal keratinocytes were changed in the skin, whereas pathways involved in hematopoetic cell lineage and immune response were found to be differentially expressed in peripheral blood.

Overall, these findings demonstrated a domino effect model of anti-TNF- *α* action in improving psoriasis, which need to further clarify at a systemic level especially using a standardized protocol for method analysis. In the current study, we performed the transcriptome analysis of blood samples from psoriatic patients treated with etanercept to characterize gene pathways activated by treatment as well as treatment response-related expression biomarkers, aiming to obtain better mechanistic insight for the further improvement of psoriasis treatment.

## Results

### Sample description

Six adult patients with moderate to severe psoriasis were treated with etanercept 50 mg subcutaneously twice weekly for 12 weeks from a clinical trial. The patients were all responders, as assessed by the change in PASI75 at 12 wk. The time period of 12 wk was chosen according to the consensus statement on biological agents for the treatment of psoriasis. The demographics and clinical information of the patients were outlined in Table 1.

**Table 1:** Characteristics of psoriasis patients included in this study

Patient ID	sex	Age	BMI	PASI	PASI12W	%PASI decrease
1	Male	64	21.6	21.6	1.2	94.4
2	Male	55	27.2	32.2	3.7	88.5
3	Male	23	20.1	24.3	2.8	88.5
4	Male	51	22	18.7	2.5	86.6
5	Male	61	27.3	43.6	6	86.2
6	Male	35	21.6	19.9	3	84.9

### Differentially-expressed gene (DEG) analysis

**Fig. 1:**
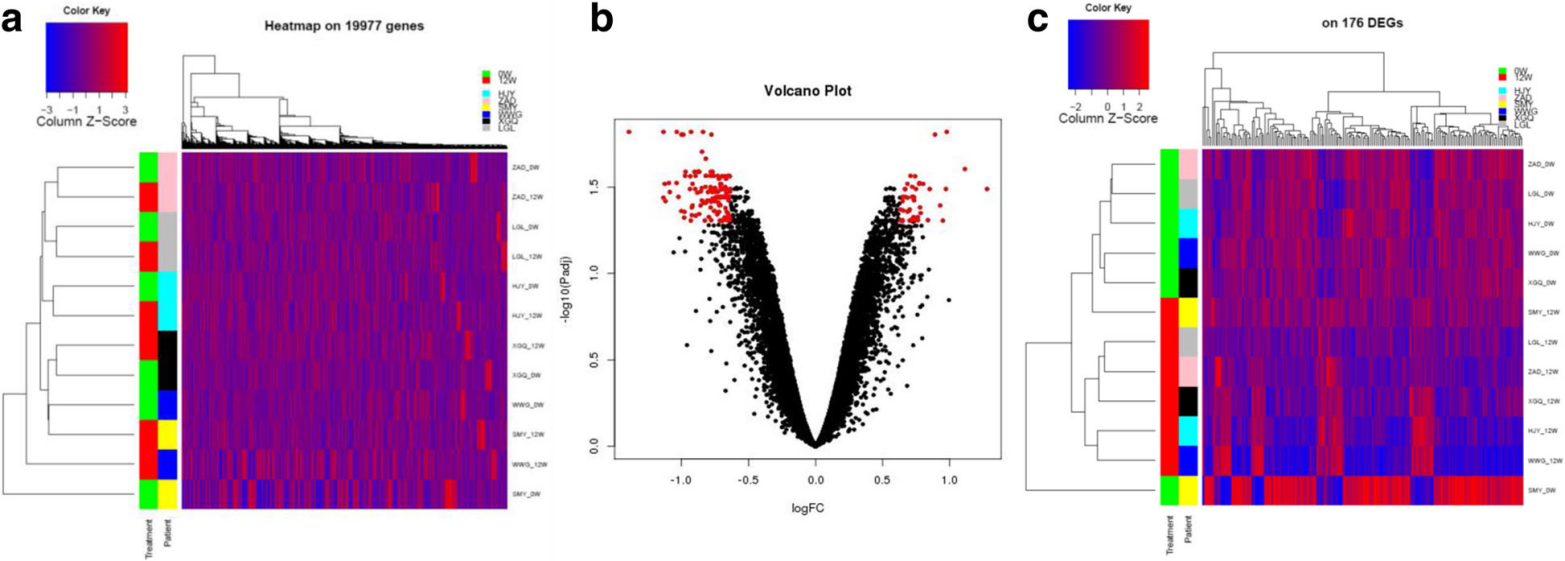
**a** The heatmap on all expressed genes; **b** The volcano plot of moderated Paired-T test, the red dots are 176 differential expressed genes DEGs with at least 1.55-fold-change between two groups of samples, before and after treatment, and BH adjusted *p*-value <0.05; **c** the heatmap on 176 DEGs

34585 probes with detected signal *p*-value < 0.05, after background correction and quantile normalization, the probe-level expression is transformed into gene-level expression by using median expression of probes for the same gene symbol. After performing quality control (see materials and methods), 19777 genes were determined to be expressed and retained for differential expression analysis. A global expression analysis of all the 19777 genes was performed by using principal component analysis (PCA). No major batch effect was observed among the samples. One sample seemed to be more distant from others as “potential outliers”, but was kept for further analyses (Fig. 1). An unsupervised cluster analysis was also performed by using all the expressed genes. The heatmap of the cluster analysis indicated that the two samples (0W and 12W) from each individual were mostly clustered together, but no separation was observed between the samples collected at the baseline and the ones collected 12 weeks after treatment (Fig. 1). Although not showing evidence for strong batch effect, the global expression pattern mainly showed the individual difference instead of treatment effect (before and after).

We then performed differential expression analysis by carrying out pair-wise comparison analysis between the 0W and 12W samples of individual patients using paired t-test. We identified 176 differential expressed genes (DEGs) (Additional file 1) between baseline and 12-week after treatment with adjusted *p*-value <0.05 and 1.55-Fold Change, of which 45 were up-regulated and 131 were down-regulated after 12-week treatment (Fig. 1). An unsupervised cluster analysis was performed by using the expression profiles of these 176 DEGs. As expected, most of the 0W and 12W samples could be clustered into different groups and thus classified correctly between the baseline and after treatment. It shows that SMY_0W is out of the two groups, which is also suggested as “potential outliers” by the global expression analysis above. In the two groups of other samples, the same patient after treatment, “SMY_12W”, is misclassified into the group before treatment. These misclassification results implicate that the individual gene signatures may be incomplete and inaccurate, therefore need to be further improved.

### Gene co-expression module and module DEG analysis

To further identify the DEGs with coherent or related molecular functions, we investigated and grouped the genes/transcripts with correlated expressions into co-expression modules using the weighed gene co-expression network analysis (WGCNA). After identifying co-expression modules, we then evaluated the correlation of these co-expression modules with treatment response. To do so, the expression profiles of all the genes within each module were first ‘summarized’ through PCA analysis, and the first vector of PCA (capturing the largest portion of expression variation of the module) was then evaluated for its correlation with treatment. We found 13 modules that are significantly positive-correlated with treatment (correlation coefficient > 0.5, *p*-value < 0.05); and 9 modules that are significantly negative-correlated with treatment (correlation coefficient < -0.5, *p*-value < 0.05) (Additional file 2). These treatment correlated modules are likely enriched for the functionally related genes whose expression are correlated with treatment, and DEGs within these modules are more likely (than other DEGs) to be the ones that are involved in etanercept therapy response.

**Fig. 2:**
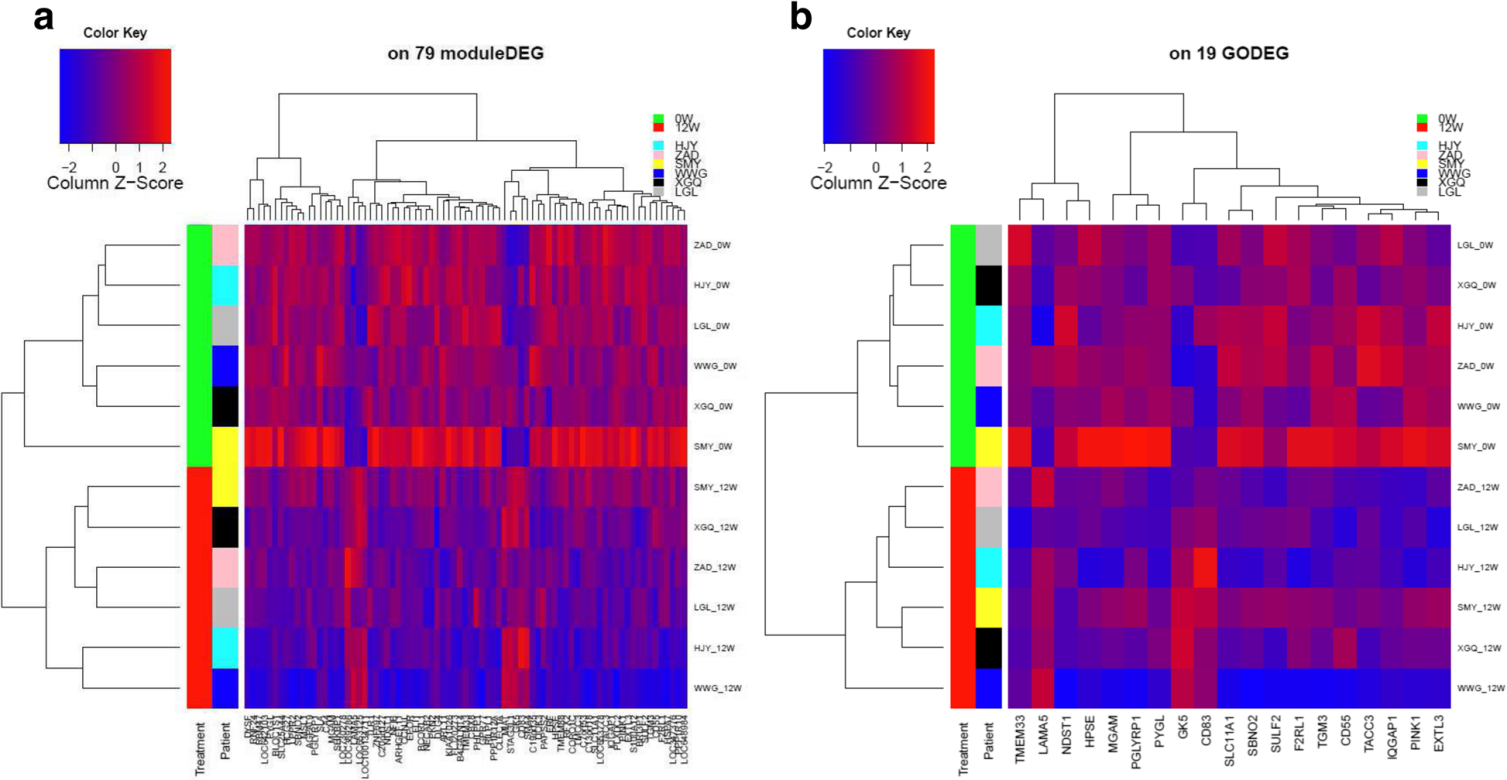
**a** The heatmap on 79 module DEGs in the significantly treatment response correlated modules; **b** The heatmap on 19 final gene markers (GO_DEG)

Among the 176 DEGs, only 79 were within these treatment correlated co-expression modules and referred to as “Module DEGs (M_DEGs)”. As expected, the unsupervised clustering analysis using these 79 M_DEGs provided better performance than the analyses by using 176 DEGs, by perfectly separating all the 0W samples from all the 12W ones (Fig. 2).

### GO enrichment analysis and GO_DEG

Last, we performed GO enrichment analysis to identify the functional processes and gene pathways that are enriched for the 79 M_DEGs. To further narrow down the pathways that play an important role in treatment response, we evaluated the correlation of the overall gene expression activity of each pathway with treatment response. About 30 pathways were found to be significantly correlated with treatment response (*p*-value < 0.01), and these pathways are likely to be the ones that are involved in treatment response (Table 2). Of these 30 pathways, 4 pathways were related to immune response and inflammation (positive regulation of CD4+ *αβ* T cell activation, myeloid cell activation involved in immune response, regulation of CD4-positive, alpha-beta T cell activation, regulation of natural killer cell differentiation), 8 were involved in polysaccharide metabolic process, 5 were involved on the skin epidermal and hair follicle development, 4 pathways were enriched to positive regulation vascular endothelial growth factor (VEGF) production, positive regulation of protein kinase B signalling, glomerulus development and chondrocyte differentiation accordingly. Interestingly, only 19 M_DEGs were within these 30 pathways, and their expression profiles can achieve the same performance as the 79 M_DEGs in term of distinguishing the samples before the treatment (baseline 0W) from the ones after the treatment (12W) (Fig. 2) and can potentially be used as biomarkers for etanercept treatment.

**Table 2:** The top enriched GO pathways (pathway size <100; enrichment *p*-value < 0.1) which also significantly correlated with treatment response (|*correlation*|> 0.7; correlation *p*-value < 0.01)

GO Pathway	Treatment response correlation	GO-Enrichment *P*value	M_DEG
Heparan sulfate proteoglycan metabolic process	0.81	2.50E-06	EXTL3, HPSE, NDST1, SULF2
Cellular carbohydrate catabolic process	0.86	0.00135	GK5, MGAM, PYGL
Positive regulation of CD4-positive, alpha-beta T cell activation	0.90	0.00268	CD55, CD83
Hair follicle development	0.80	0.00273	HPSE, LAMA5, TGM3
Molting cycle process	0.80	0.00273	HPSE, LAMA5, TGM3
Hair cycle process	0.80	0.00273	HPSE, LAMA5, TGM3
Skin epidermis development	0.80	0.00283	HPSE, LAMA5, TGM3
Positive regulation vascular endothelial growth factor production	0.74	0.00317	HPSE, SULF2
Glucan catabolic process	0.88	0.00369	MGAM, PYGL
Cellular polysaccharide catabolic process	0.88	0.00397	MGAM, PYGL
Regulation of vascular endothelial growth factor production	0.79	0.00425	HPSE, SULF2
Polysaccharide catabolic process	0.88	0.00454	MGAM, PYGL
Vascular endothelial growth factor production	0.76	0.00485	HPSE, SULF2
Proteoglycan biosynthetic process	0.76	0.01521	EXTL3, NDST1
Myeloid cell activation involved in immune response	0.78	0.01734	F2RL1, SBNO2
Glycosaminoglycan catabolic process	0.76	0.01789	HPSE, PGLYRP1
Cellular glucan metabolic process	0.75	0.03104	MGAM, PYGL
Glucan metabolic process	0.75	0.03104	MGAM, PYGL
Glomerular epithelial cell development	0.88	0.03312	IQGAP1
Positive regulation of mitochondrial fission	0.79	0.03312	PINK1
Manganese ion transport	0.73	0.03961	SLC11A1
Regulation of hair follicle development	0.78	0.04284	HPSE
Cellular polysaccharide metabolic process	0.72	0.04295	MGAM, PYGL
Nucleus localization	0.76	0.07144	TACC3
Glycogen catabolic process	0.88	0.08388	PYGL
Peptide cross-linking	0.74	0.09616	TGM3
Cell proliferation in forebrain	0.85	0.09616	TACC3
Regulation of CD4-positive, alpha-beta T cell activation	-0.90	0.00485	CD55, CD83
Aminoglycan catabolic process	-0.75	0.02074	HPSE, PGLYRP1
Regulation of oxidative phosphorylation	-0.76	0.03312	PINK1
Nuclear pore organization	-0.80	0.03637	TMEM33
Regulation of natural killer cell differentiation	-0.71	0.03961	PGLYRP1
Pore complex assembly	-0.72	0.03961	TMEM33
Glycoprotein catabolic process	-0.77	0.04284	HPSE

## Discussion

We performed gene expression analysis in the blood samples from psoriatic patients treated with etanercept. By comparing the samples collected at the baseline (before the treatment) and the ones collected 12 weeks after the treatment, we were able to identify 176 DEGs whose expression changes are correlated with treatment response. In addition, we integrated the results of deferential expression analysis with the co-expression analysis and further identified 79 DEGs (of the 176 DEGs) that shared coherent molecular functionalities (as suggested by their co-expression patterns). With coherent functionalities and significant correlation with treatment response, these 79 DEGs likely play an important role in etanercept treatment by mediating therapeutic response through gene expression alteration. As a result, the expression profile of these genes can clearly distinguish the blood samples collected before the treatment from the ones collected after treatment (12 weeks). Furthermore, by focusing on the GO pathways whose expression profile was correlated with treatment response, we could identify a small group of 19 DEGs whose expression profile can provide perfect separation between the blood samples collected before and after the treatment. These genes can be applied as expression biomarkers for markers of the anti-TNF- *α* treatment response in psoriatic patients.

In the current study, we pursued the integration of differential gene expression and co-expression analysis to identify the genes and pathways that mediate treatment response. The integration is implemented to reduce the false positives from single view and find the consistent evidence. As co-expression modules cluster the genes with similar expression patterns, these genes are more likely (than others) to share coherent functions. Using these co-expression modules as functional filters, we were able to prioritize the DEGs with coherent functions. As we demonstrated, 79 co-expression modules related DEGs performed a better job than the total 176 DEGs in term of separating the samples before and after the treatment, suggesting that the functional coherence is a good constraint that can be used to narrow down genes whose expression alterations molecularly mediate the therapeutic response to anti-TNF- *α* treatment.

Using the 79 M_DEGs as seed, we were able to identify the pathways that are enriched for the genes and further identified the enriched pathways whose expression activities are correlated with anti-TNF- *α* treatment. Many of these pathways TNF- *α* related ones, such as skin epidermis development and molting cycle process and immune related processes, such as positive regulation of CD4-positive, alpha-beta T cell activation, myeloid cell activation involved in immune response and regulation of natural killer cell differentiation. These are well expected due to inhibition effect of the treatment on TNF- *α* activity.

In addition, our analyses also identified some novel pathways and/or functional processes, such as polysaccharide catabolic process and regulation VEGF production. Among the 6 pathways of polysaccharide catabolic process, the heparan sulfate proteoglycan (HSPG), belong to a heterogeneous family of the proteoglycans, are important components of the basement membrane (BM) of various tissues. Differential expression of HSPG has been shown in psoriatic lesions (comparing with normal skin) as well as chronically inflamed synovium from arthritis patients (comparing to normal individuals) [13, 14]. Several studies suggested their role in adhesion, cell-extracellular matrix interactions, migration, keratinocyte proliferation and differentiation, inflammation, and wound healing. There was also evidence that HSPG was involved in angiogenesis that might be related to the interaction of the HSPG with VEGF. Angiogenesis has a crucial role in the pathogenesis of psoriasis. As the most potent angiogenic factor, VEGF is directly implicated in influencing both skin lesions and systemic involvement in psoriasis and is a potential target for the treatment of psoriasis. It was significantly up-regulated in psoriatic skin lesions as compared with uninvolved skin in psoriasis patients and normal controls. Besides, Serum level of VEGF was significantly elevated in patients and correlated with clinical severity of psoriasis [15]. In addition, systemic anti-VEGF treatment strongly reduces skin inflammation in a mouse model of psoriasis [16]. Therefore, it is likely that a kind of antipsoriatic mechanism of TNF- *α* inhibitors is inhibition of angiogenesis by preventing the TNF-induced upregulation of VEGF(by reducing the synthesis of VEGF). Previous studies had also showed VEGF down-regulation in psoriatic skin lesions and in cutaneous mesenchymal stem cells from psoriasis patients responding to the anti-TNF therapy [9, 17]. In our study, we identified 3 pathways involved in VEGF production increase the expression activity after 12 weeks of anti-TNF- *α* treatment, which implied the dual role of anti-TNF- *α* therapy including inhibition of inflammation and angiogenesis. Although novel and molecularly interesting, these pathways/functional processes do need to be further investigated regarding to their involvement in mediating the treatment effect of etanercept on psoriasis.

Furthermore, we found 19 GO_DEGs within these 30 pathways. Interestingly, these 19 DEGs perform as well as 79 M_DEGs on discriminating the samples before and after the treatment. These genes can therefore be applied as expression biomarkers for the anti-TNF- *α* treatment response in psoriatic patients. Among them, CD83 and SLC11A1 are enriched in several pathways related to immune response and inflammation. CD83 is a marker for mature/activated dendritic cells, which are a major source of TNF in psoriasis lesions and provide signals for direct intralesional T cell activation. We found that CD83 were up-regulated after 12-week treatment, which is not with the finding from the previous study by Gottlieb et al. [18] where decreased CD83 mRNA expression was observed in psoriatic skin lesions after 12-week treatment of etanercept. Solute Carrier Family 11A Member 1 (SLC11A1), also known as natural resistance-associated macrophage protein 1(NRAMP1), has an immunoregulatory role in macrophage activation and the Th1/Th2 balance of the adaptive immune response to infection. Previous studies have showed that genetic variants in SLC11A1 gene are associated with the susceptibility to autoimmune diseases such as rheumatoid arthritis and multiple sclerosis, and infectious diseases including tuberculosis and leprosy [19]. So, it suggested these genes might be novel causal genes in related to immune response of etanercept treatment in psoriasis. We’ll collect more clinical samples for further validation study on these novel findings.

## Conclusions

In summary, through an integrated analysis of differential expression and co-expression, we have explored the application of functional coherence (co-expression in the current study) in transcriptome analysis and identified both known and novel anti-TNF- *α* treatment based novel function. Our result suggests that is a good constraint that can be used to narrow down genes whose expression alterations molecularly mediate the therapeutic response to anti-TNF- *α* treatment.

## Methods

### Sample description

Six adult patients with moderate to severe psoriasis who were going to be treated with etanercept 50 mg subcutaneously twice weekly were recruited for this study. Patients were excluded if they had already received any systemic or local medications 4 weeks before inclusion. Before and during treatment, patients were evaluated clinically with Psoriasis Area Severity Index (PASI) score. The time period of 12 wk was chosen according to the consensus statement on biological agents for the treatment of psoriasis. The patients were all responders, as assessed by the change in PASI75 at 12 wk. The demographics and clinical information of the patients were outlined in Table 1. All patients provided signed informed consent for participating in this study. The study was approved by the local institutional ethics review board, the 2 ^*nd*^ affiliated hospital, Zhejiang University school of medicine.

### RNA microarray data collection

The blood samples before etanercept treatment (0W) and 12 weeks (12W) after treatment of drug etanercept were collected from 6 psoriasis patients. Total RNA was extracted and purified from these 12 blood samples by using QIAGEN ^*Ⓡ*^ QIAamp RNA Blood Mini Kit according to the manufacturer’s instructions. After QC analysis, 500ng total RNA was amplified using Illumina^Ⓡ^ TotalPrep^TM^ RNA Amplification Kit (Ambion). Amplified RNA was hybridized overnight with HumanHT-12 v4 Expression BeadChips (Illumina) according to manufacturer’s protocols. The slides were scanned by iSCAN. The Illumina GenomeStudio software generates the bead summary data with a single signal intensity value for each probe.

### Background correction and normalization

The background correction was done in GenomeStudio by subtracting the mean signal of negative control probes [20] in a particular array from each bead summary value in that array. To avoid distortion of the results by noise due to background signal rather than specific probe-target hybridization, the specificity of individual probe signals was estimated using the detection *p*-value, which is the probability of seeing a certain signal level without probe-target hybridization. All the probes returning a detection *p*-value > 0.05 in both the control group and the case group were eliminated from further analysis. The quantile normalization was implemented by using “neqc” function in R package “limma” [21].

### Gene-level differential expression test

As we focused on gene-level expression analysis, the expression level of a particular gene was calculated by using the median expression of all the probes within this gene. The low-expressed genes are filtered out by R package “genefilter” to make the remaining genes are those expressed at least 3 samples among the 12 samples.

Assuming the independence among genes, we used a linear model with sample-pair effects for each gene to fit the expression data by “lmFit” from “limma” [21]. After fitting the linear additive model, the limma moderated paired t-test (the moderated t statistic is modifying t-statistic by adding small constant to the pooled standard deviation to avoid divisions by an extremely small variance estimate, suitable for small sample size with small variance on some genes) of differential expression for each probe was implemented by “eBayes”. The empirical Bayes analysis borrows the information across probes to improve power for small sample size by shrinking the probe-wise sample variances towards a common value [22].

### Treatment response correlated co-expression modules from WGCNA

Correlated expressions of different genes (co-expression) suggest shared molecular functions/gene pathways. To capture such global co-expression (functional coherent) modules from gene expression profiles, we performed weighted gene co-expression network analysis (WGCNA) by using the R package, “WGCNA” [23], which identify co-regulated gene sets based on correlation of gene pairs and assign sets of co-regulated genes to co-expression modules via hierarchical clustering. The minimum module size is set as 30.

We further attempted to further narrow down the co-expression modules whose expression activities are associated with the treatment response. First, we performed the PCA analysis using the expression data of all the genes within a particular co-expression module, and the overall expression activity of a module (eigengene expression) was estimated by using the value of the first principle component of PCA analysis. Then, we calculated the Pearson correlation of the eigengene expression of each module with treatment by coding the samples before treatment (0W) as “1” and the ones after treatment (12W) as “2” and estimated the significance of correlation (p vlaue) using student t-test. The significant treatment-correlated modules are the modules whose absolute correlations with traits are above 0.5 and corresponding *p*-value <0.05, that is, significantly correlated with the traits. Note that the positive correlation with “12W” means up-regulation of overall expression activity with treatment, and the negative correlation means down-regulation of overall expression activity with treatment.

### Treatment-correlated top pathways that are enriched for module DEGs

The GO pathways enrichment was implement by an R package,“topGO” [24], where hypergeometric enrichment *p*-value is reported. We didn’t report the FDR adjusted *p*-value here as it’s too conservative to get very few GO pathways as significant [24]. To not omit the interesting GO pathways, we first used enrichment *p*-value to filter the GO pathways, and then used the pathway correlation with treatment traits to reduce the false positive discovery rate.

**Fig. 3:**
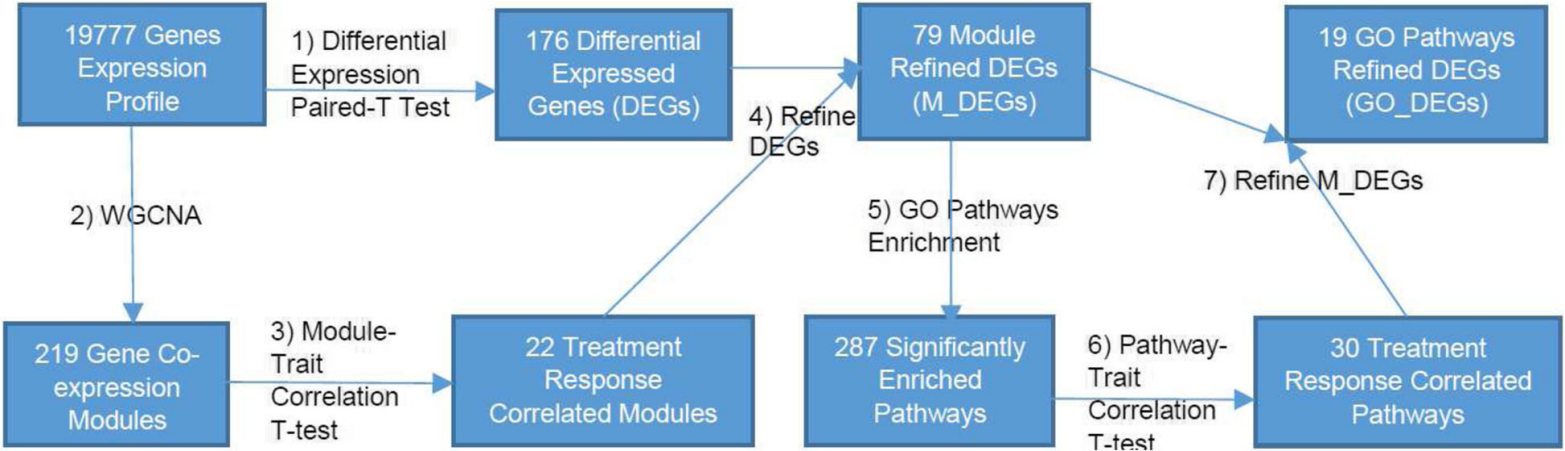
The whole analysis procedures: 1) 176 DEGs are obtained by paired-t test of 19777 genes expression of patients before and after treatment; 2) 219 gene co-expression modules are obtained by Weighted Gene Co-expression Network Analysis (WGCNA) [23], 3) 22 gene co-expression modules are significant correlated with treatment response traits; 4) 79 DEGs are refined by the 22 treatment response correlated modules as M_DEGs; 5) 287 GO pathways are significantly enriched (pathway size < 100 and *p*-value < 0.1) from 79 M_DEGs; 6) 30 GO pathways gene expression activity are significantly correlated with treatment response traits (|*correlation*|> 0.7; correlation *p*-value < 0.01); 7) 19 M_DEGs are refined by the 30 treatment response correlated GO pathways as GO_DEGs

The specific GO pathways whose pathway size (the number of the gene members in the pathway) is less than 100, were enriched from the module refined DEGs (M_DEGs), and the significant ones with enrichment *p*-value less than 0.1 were selected. For these significant enriched pathways, their expression correlation with treatment traits were also investigated by the same procedures as what we did for the co-expression module in the above section. The significantly treatment traits correlated GO pathways (Pearson correlation above 0.7 or below −0.7; Pearson correlation *p*-value less than 0.01) were selected as treatment response correlated pathways, which were thus most likely the ones that mediate the anti-TNF- *α* treatment response through transcriptional alteration. Lastly, we identified the DEGs within these correlated GO pathways as GO_DEGs. The whole work flow of the data analyses procedures is shown in Fig. 3.

## Additional files

Additional file 1 Additional Table 1. 176 differential expressed genes (DEGs) between baseline and 12-week after treatment with adjusted *p*-value <0.05 and 1.55-Fold Change by using paired t-test. (XLSX 20 kb)

Additional file 2 Additional Table 2. The details of co-expression modules generated from WGCNA, where 13 modules that are significantly positive-correlated with treatment (correlation coefficient > 0.5, *p*-value < 0.05); and 9 modules that are significantly negative-correlated with treatment (correlation coefficient < -0.5, *p*-value < 0.05). (XLSX 375 kb)

## Abbreviations

DEG: Differentially-expressed gene
PCA: Principal components analysis
PBMC: Peripheral blood mononuclear cell
TNF: Tumor necrosis factor
WGCNA: Weighed gene co-expression network analysis

## References

[1] Crow JM (2012). Psoriasis uncovered. Nature.

[2] Kim J, Krueger JG (2015). The immunopathogenesis of psoriasis. Dermatol Clin.

[3] Schottelius AJ, Moldawer LL, Dinarello CA, Asadullah K, Sterry W, Edwards III CK (2004). Biology of tumor necrosis factor-alpha- implications for psoriasis. Exp Dermatol.

[4] Zaba LC, Suarez-Farinas M, Fuentes-Duculan J, Nograles KE, Guttman-Yassky E, Cardinale I, Lowes MA, Krueger JG (2009). Effective treatment of psoriasis with etanercept is linked to suppression of IL-17 signaling, not immediate response TNF genes. J Allergy Clin Immunol.

[5] Balato A, Schiattarella M, Di Caprio R, Lembo S, Mattii M, Balato N, Ayala F (2014). Effects of adalimumab therapy in adult subjects with moderate-to-severe psoriasis on Th17 pathway. J Eur Acad Dermatol Venereol.

[6] Brunner PM, Koszik F, Reininger B, Kalb ML, Bauer W, Stingl G (2013). Infliximab induces downregulation of the IL-12/IL-23 axis in 6-sulfo-lacnac (slan)+ dendritic cells and macrophages. J Allergy Clin Immunol.

[7] Johnston A, Guzman AM, Swindell WR, Wang F, Kang S, Gudjonsson JE (2014). Early tissue responses in psoriasis to the antitumour necrosis factor-alpha biologic etanercept suggest reduced interleukin-17 receptor expression and signalling. Br J Dermatol.

[8] Wang F, Smith N, Maier L, Xia W, Hammerberg C, Chubb H, Chen C, Riblett M, Johnston A, Gudjonsson JE (2012). Etanercept suppresses regenerative hyperplasia in psoriasis by acutely downregulating epidermal expression of interleukin (IL)-19, IL-20 and IL-24. Br J Dermatol.

[9] Bose F, Petti L, Diani M, Moscheni C, Molteni S, Altomare A, Rossi RL, Talarico D, Fontana R, Russo V (2013). Inhibition of ccr7/ccl19 axis in lesional skin is a critical event for clinical remission induced by tnf blockade in patients with psoriasis. Am J Pathol.

[10] Johansen C, Vinter H, Soegaard-Madsen L, Olsen LR, Steiniche T, Iversen L, Kragballe K (2010). Preferential inhibition of the mRna expression of p38 mitogen-activated protein kinase regulated cytokines in psoriatic skin by anti-TNF *α* therapy. Br J Dermatol.

[11] Bose F, Raeli L, Garutti C, Frigerio E, Cozzi A, Crimi M, Caprioli F, Scavelli R, Altomare G, Geginat J (2011). Dual role of anti-TNF therapy: enhancement of TCR-mediated T cell activation in peripheral blood and inhibition of inflammation in target tissues. Clin Immunol.

[12] Chow M, Lai K, Ahn R, Gupta R, Arron S, Liao W (2016). Effect of adalimumab on gene expression profiles of psoriatic skin and blood. J Drugs Dermatol.

[13] Patterson AM, Cartwright A, David G, Fitzgerald O, Bresnihan B, Ashton BA, Middleton J (2008). Differential expression of syndecans and glypicans in chronically inflamed synovium. Ann Rheum Dis.

[14] Seyger MM, van den Born J, Schalkwijk J, van de Kerkhof PC, de Jong EM (1997). Altered distribution of heparan sulfate proteoglycans in psoriasis. Acta Derm Venereol.

[15] Bhushan M, McLaughlin B, Weiss JB, Griffiths CE (1999). Levels of endothelial cell stimulating angiogenesis factor and vascular endothelial growth factor are elevated in psoriasis. Br J Dermatol.

[16] Schonthaler HB, Huggenberger R, Wculek SK, Detmar M, Wagner EF (2009). Systemic anti-VEGF treatment strongly reduces skin inflammation in a mouse model of psoriasis. Proc Natl Acad Sci.

[17] Campanati A, Orciani M, Ganzetti G, Consales V, Di Primio R, Offidani A (2016). The effect of etanercept on vascular endothelial growth factor production by cutaneous mesenchymal stem cells from patients with psoriasis. J Int Med Res.

[18] Gottlieb AB, Chamian F, Masud S, Cardinale I, Abello MV, Lowes MA, Chen F, Magliocco M, Krueger JG (2005). TNF inhibition rapidly down-regulates multiple proinflammatory pathways in psoriasis plaques. J Immunol.

[19] Awomoyi AA (2007). The human solute carrier family 11 member 1 protein (SLC11A1): linking infections, autoimmunity and cancer?. FEMS Immunol Med Microbiol.

[20] Shi W, Oshlack A, Smyth GK (2010). Optimizing the noise versus bias trade-off for illumina whole genome expression beadchips. Nucleic Acids Res.

[21] Ritchie ME, Phipson B, Wu D, Hu Y, Law CW, Shi W, Smyth GK (2015). limma powers differential expression analyses for RNA-sequencing and microarray studies. Nucleic Acids Res.

[22] Smyth GK (2004). Linear models and empirical bayes methods for assessing differential expression in microarray experiments. Stat Appl Genet Mol Biol.

[23] Langfelder P, Horvath S (2008). WGCNA, an R package for weighted correlation network analysis. BMC Bioinformatics.

[24] Alexa A, Rahnenfuhrer J. Gene set enrichment analysis with topGo. 2018. (www.bioconductor.org).

